# Limits of Predicting Colorectal Cancer PDX Engraftment: A Specimen-Grouped Machine Learning Analysis of 1007 Establishment Attempts

**DOI:** 10.3390/cancers18182996

**Published:** 2026-09-16

**Authors:** Menglan Liu, Mathias Krohn, Sandra Schwarz, Maria Witte, Friedrich Prall, Michael Linnebacher

**Affiliations:** 1 Molecular Oncology and Immunotherapy, Clinic of General Surgery, University Medical Center Rostock, Schillingallee 35, 18057 Rostock, Germany; liumenglan01@outlook.com (M.L.); mathias.krohn@med.uni-rostock.de (M.K.); sandra.schwarz@med.uni-rostock.de (S.S.); maria.witte@med.uni-rostock.de (M.W.); 2 Institute of Pathology, University Medical Center Rostock, 18057 Rostock, Germany; friedrich.prall@web.de

**Keywords:** colorectal cancer, patient-derived xenograft, engraftment success, machine learning, grouped cross-validation, predictive modeling

## Abstract

**Background:** Patient-derived xenograft (PDX) models retain clinically relevant features of the donor tumor, but the establishment of colorectal cancer (CRC) PDX models succeeds in only a fraction of attempts. Whether routinely available clinicopathological and experimental variables can predict engraftment for a previously unseen tumor has not been established under a validation design that respects the clustered structure of PDX datasets. **Methods:** We retrospectively analyzed 1007 first-generation CRC-PDX establishment attempts from 381 specimens (328 patients). Primary analysis used specimen-grouped validation with nested grouped cross-validation; all preprocessing was performed within training folds. Secondary analyses included patient-grouped, attempt-level, specimen-level and temporal validation. **Results:** Attempt success was 46.3%; 68.8% of specimens yielded ≥1 PDX. Within-specimen correlation was substantial (0.31). Under specimen-grouped validation, pooled AUC was 0.550 (95% CI 0.508–0.591), PR-AUC was 0.500, Brier was 0.253, and sensitivity/specificity was 0.42/0.64. Patient-grouped validation was similar (0.548), while attempt-level splitting inflated AUC to 0.68–0.69, confirming bias from specimen clustering rather than preprocessing leakage. Specimen-level prediction reached 0.636; calendar-time temporal validation yielded 0.584. Sensitivity analyses were stable. **Conclusions:** Routine variables offer only marginal discrimination for CRC-PDX engraftment in new tumors. Moderate attempt-level performance reflects repeated sampling of the same tumors, not a generalizable signal. These results do not support routine-variable-based triage and identify molecular and tissue-quality factors as the necessary next step.

## Simple Summary

Patient-derived xenograft (PDX) models are grown by implanting fragments of a patient’s tumor into immunodeficient mice, but only about half of such attempts produce a usable model. We asked whether the information routinely recorded about a tumor and about the implantation procedure is sufficient to predict, in advance, which tumors will engraft. Using 1007 implantation attempts performed over ten years, we show that the answer depends critically on how a prediction model is tested. When attempts from the same tumor are allowed to appear in both the training and the test data, models appear to perform well. When each tumor is kept entirely within either the training or the test set—the situation that corresponds to predicting the fate of a genuinely new tumor—discrimination falls to only slightly better than chance. Routinely available clinical, pathological and procedural variables are therefore not sufficient to triage tumors before implantation, and the determinants of engraftment must be sought in molecular and tissue-quality data that are not currently collected.

## 1. Introduction

Colorectal cancer (CRC) is the third most commonly diagnosed malignancy worldwide and remains a leading cause of cancer-related mortality [1]. Despite advances in screening and therapeutic strategies, clinical outcomes remain poor owing to tumor heterogeneity, treatment resistance, and complex disease biology [2,3,4,5,6].

Patient-derived xenograft (PDX) models have become an important platform for translational cancer research [7,8,9,10,11,12]. By directly implanting patient tumor tissues into immunodeficient mice, PDX models preserve key histopathological and molecular characteristics of the original tumors, including intratumoral heterogeneity, thereby providing a biologically relevant system for studying tumor progression and therapeutic response [13,14].

Despite their broad application, the successful establishment of PDX models remains a major challenge. Reported engraftment rates range from approximately 20% to 70% across studies. Previous investigations have suggested that both tumor-intrinsic factors and experimental variables may influence engraftment success. Factors such as tumor stage, histological grade, metastatic status, molecular subtype, and specimen handling procedures have all been proposed as potential determinants [15,16,17,18,19]. However, findings remain inconsistent across studies, and no consensus has emerged regarding which variables are most strongly associated with successful PDX establishment.

To address this gap, we therefore retrospectively analyzed 1007 first-generation CRC-PDX establishment attempts accumulated over a ten-year period and evaluated the predictive value of routinely available clinicopathological and experimental variables under a specimen-grouped validation design as the primary analysis, with attempt-level, patient-level, specimen-level and temporal designs compared directly in the same dataset.

## 2. Materials and Methods

### 2.1. Ethical Approval and Study Registration

The study was conducted in accordance with the principles outlined in the Declaration of Helsinki. Informed written consent was obtained from all participating patients. Ethical approval was granted by the Ethics Committee of the University Medical Center Rostock (protocol numbers: II HV 43/2004, A45/2007, A2018-0054, and A2019-0187) for the use of human tissue.

### 2.2. Patients and Tumor Tissue Collection

Eligible patients were those who presented with primary, metastatic, or recurrent CRC and underwent treatment at the University Medical Center Rostock between July 2014 and July 2024. Inclusion required a histopathological diagnosis of CRC and an age of at least 18 years with surgical resection or large-scale biopsy. All enrolled patients provided written informed consent to participate in the study and to donate tumor tissue. Exclusion criteria were as follows: a history of other malignant tumors of the abdomen, thorax, or cranium within the preceding three years; severe comorbidities likely to compromise surgery or tissue collection; and inability or unwillingness to give informed consent. Tumor tissue was collected during routine surgical or laparoscopic procedures following the standard operating procedures of the BioBank Rostock. Fresh tumor tissue was promptly transported to the laboratory for processing, cryopreservation, or xenotransplantation into immunodeficient mice; the remaining material was forwarded to pathology for histopathological and molecular characterization.

### 2.3. Animal Experiments

All animal experiments were conducted in accordance with the established guidelines for animal care and use. They were in compliance with institutional and local regulatory standards as authorized by the Landesamt für Landwirtschaft, Lebensmittelsicherheit und Fischerei Mecklenburg-Vorpommern under permit numbers LALLF M-V/TSD/7221.3-1.1-071/10, 7221.3-1-015/14, 7221.3-2-020/17, and 7221.3-2-023/20.

All experimental mice used in this study were obtained from the Rostock Animal Center. The mouse strains employed included NMRI-Foxn1nu/nu (NMRI nude), NOD.Cg-Prkdcscid Il2rgtm1Wjl/SzJ (NSG), and NOD.Cg-Prkdcscid (NOD SCID). All mice were maintained under specific pathogen-free conditions. The mice were of mixed sex, aged between 6 and 8 weeks, and weighed between 18 and 30 g. They were housed under strictly controlled environmental conditions with a temperature of 22 °C ± 2 °C, humidity maintained at 50–60%, and a 12 h light–dark cycle. Standard laboratory chow was provided daily, along with ad libitum access to clean drinking water.

### 2.4. Procedure of PDX Model Establishment and Harvest

PDX generation has been described in detail previously [20]. Briefly, fresh tumor tissue pieces (3 mm × 3 mm × 3 mm), or pieces stored viably at −150 °C, were used for xenografting. A subset of pieces was soaked in 100 µL Matrigel (Corning, Kaiserslautern, Germany) for at least 10 min at 4 °C prior to subcutaneous implantation into the flanks of anesthetized mice (ketamine/xylazine, 90/6 mg/kg body weight). Each mouse constituted a single establishment attempt irrespective of whether tumor fragments were implanted into one or both flanks; engraftment outcomes were therefore recorded at the level of the individual animal. Postoperatively, mice received drinking water supplemented with antibiotics (cotrimoxazole: 8 mg trimethoprim and 40 mg sulfamethoxazole per kg body weight) for six weeks to prevent infection. Tumor growth was monitored every two days. Tumor volume was calculated as V = 0.5 × Dmax × Dmin^2^, where Dmax is the maximum and Dmin is the minimum tumor diameter. At harvest, PDX tumor samples were cut into pieces and rapidly frozen in liquid nitrogen (4 mm × 4 mm × 4 mm) for subsequent molecular analyses. For preservation of the respective models, four viable 3 mm × 3 mm × 3 mm tumor pieces were transferred into one 2 mL cryotube containing 1.5 mL freezing medium (fetal bovine serum with 10% DMSO) and gradually cooled to −80 °C using a CoolCell^®^ LX freezing container (CryoShop, Munich, Germany) at 1 °C per minute.

### 2.5. Histopathology

Representative tumor samples were fixed in 10% neutral-buffered formalin, paraffin-embedded, sectioned (4–5 μm), and stained with hematoxylin and eosin. Histological evaluation was performed by an experienced pathologist, and the morphology of each PDX tumor was compared with its corresponding patient tumor to confirm histological consistency [21].

### 2.6. Short Tandem Repeat (STR) Analysis

STR analysis was employed to confirm the identity of PDX tumor samples relative to patient tumors. Briefly, genomic DNA was first extracted from tissue samples using column-based methods with commercial kits. The extracted DNA was then PCR-amplified for specific short tandem repeat regions, including D5S818, D7S820, D16S539, D13S317, vWA, TPOX, THO1, CSF1PO, and Amelogenin, according to standard procedures in a multiplex reaction. The results were compared between PDX and patient tissue to confirm the identity of the PDX samples with the original patient tumors.

### 2.7. Assessment of the Success of PDX Models

**Figure 1:**
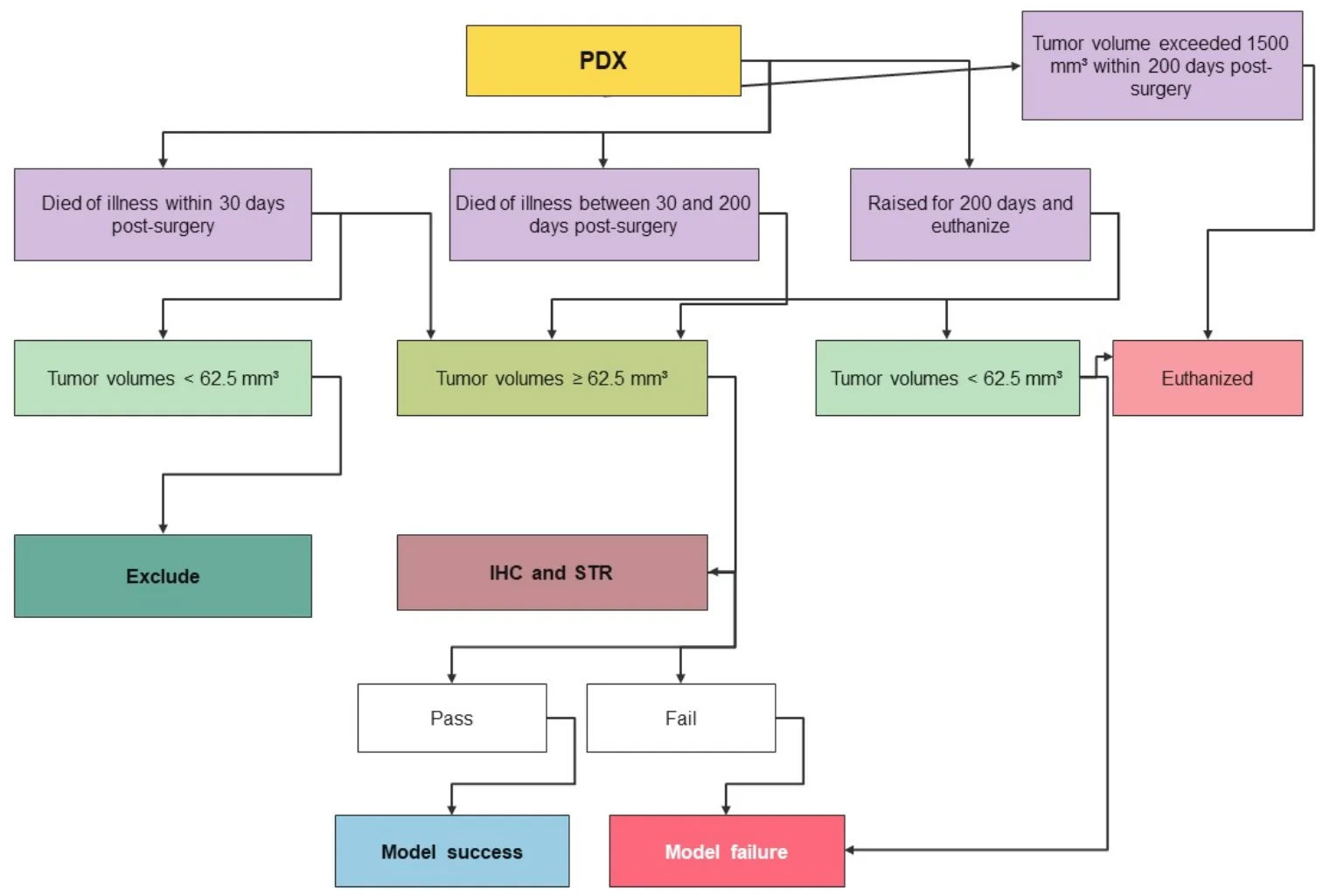
Assessment of the success of PDX models. Flowchart illustrating the criteria used to classify first-generation CRC-PDX establishment attempts as successful or unsuccessful. Tumor growth, survival time, tumor volume, histopathological examination and STR analysis were used to determine the final outcome of each attempt.

Experimental mice were euthanized via CO_2_ asphyxiation followed by cervical dislocation according to institutional guidelines when the tumor volume exceeded 1500 mm^3^ after surgery. For mice that died from illness within 200 days after surgery with tumor volumes ≥ 62.5 mm^3^ (D_min_ ~ ≥ 5 mm), tumor samples were collected for IHC and STR confirmation. Cases in which mice died within 30 days of surgery with tumor volumes < 62.5 mm^3^ were excluded. Mice with tumor volumes < 62.5 mm^3^ at 200 days after surgery were euthanized and classified as failures without further analysis. Detailed criteria for determining PDX success and failure are provided in Figure 1. The 62.5 mm^3^ cut-off corresponds to a minimum tumor diameter of approximately 5 mm, the smallest size at which a subcutaneous nodule can be reliably distinguished by palpation and caliper measurement from post-operative changes; below this size, a lesion cannot be confirmed as a growing tumor.

### 2.8. Predictors and Missing Data

To investigate factors associated with CRC-PDX establishment, a machine-learning framework was developed using 1007 first-generation PDX modeling attempts. Clinical, pathological, and experimental variables were included as candidate predictors. Clinical variables comprised patient age and sex; pathological variables included tumor grade, UICC stage, MSI status, and tumor location; and experimental variables reflected modeling procedures and host-related characteristics.

Missing values were confined to four variables: tumor grade (12.4%), lymphatic invasion (13.1%), vascular invasion (13.5%) and MSI status (20.3%). All other predictors were complete. MSI results recorded only as “MSI” without specification of high or low instability were treated as missing, consistent with the unknown category reported in Table 1. Complete missing data percentages for every predictor are given in Supplementary Table S1.

Missing values were imputed by iterative random-forest imputation. Critically, the imputation model was fitted on the training fold of each resampling iteration only and applied unchanged to the held-out fold; no information from held-out specimens entered the imputation, standardization, feature-selection or tuning steps at any point. Three prespecified sensitivity analyses addressed the imputation strategy: median imputation, treatment of missingness as an explicit category, and a complete-case analysis.

### 2.9. Model Development and Validation

The order of operations within every resampling iteration was as follows: (1) partition the data, respecting the grouping variable; (2) fit the imputation model on the training fold and apply it to both folds; (3) fit standardization parameters on the training fold and apply them to both folds; (4) rank features by random-forest importance computed on the training fold only and retain the top ten; (5) select hyperparameters by nested grouped cross-validation within the training fold (three inner folds grouped by the same variable as the outer split, grid over the number of variables sampled per split and the minimum node size); (6) fit the final model on the whole training fold; (7) evaluate once on the held-out fold. This sequence was repeated over 100 resampling iterations with an 80/20 partition.

The primary analysis is grouped by tumor specimen. In the deposited dataset, each specimen is identified by its HROC accession code, and multiple specimens from the same patient share the same numeric stem with distinguishing suffixes (for example, “Met1” or “Tu2”); patient-level grouping was reconstructed from this stem, yielding 328 patients across the 381 specimens. Prespecified secondary analyses used the identical pipeline while varying only the partitioning unit or the outcome: grouping by patient; random attempt-level splitting with the same leakage-free pipeline; random attempt-level splitting with imputation, standardization and feature selection performed once on the full dataset, reproducing the conventional but optimistic protocol; and a specimen-level analysis in which the 381 specimens formed the units. The outcome was whether the specimen yielded at least one model; the number of attempts was included as a predictor, and partitioning was grouped by patient.

Performance was summarized as the mean test AUC across iterations with the 2.5th and 97.5th percentiles of the iteration-specific values. Because iterations are not independent, formal confidence intervals were derived separately by pooling out-of-fold predictions across iterations (averaging predictions for each attempt over the iterations in which it was held out); the resulting interval accounts for within-specimen correlation. Evidence that discrimination exceeded chance was assessed by whether this cluster-bootstrap interval excluded 0.50. Precision–recall AUC, Brier score, calibration by deciles of predicted risk, and sensitivity, specificity, and positive and negative predictive values at both a 0.5 threshold and the Youden-optimal threshold were reported for the pooled out-of-fold predictions. Feature-selection stability was quantified as the proportion of resampling iterations in which each variable was retained.

To describe associations without conflating them with predictive performance, generalized estimating equations with a binomial family, logit link, exchangeable working correlation and specimen-level clustering were fitted univariably and multivariably; the resulting odds ratios are reported with cluster-robust confidence intervals and are interpreted as associations, not as causal effects.

Descriptive statistics are presented as mean ± standard deviation for continuous variables and as frequencies and percentages for categorical variables. Analyses were performed in R version 4.4.2 and Python 3.12.

## 3. Results

### 3.1. Clinicopathological Characteristics

**Figure 2:**
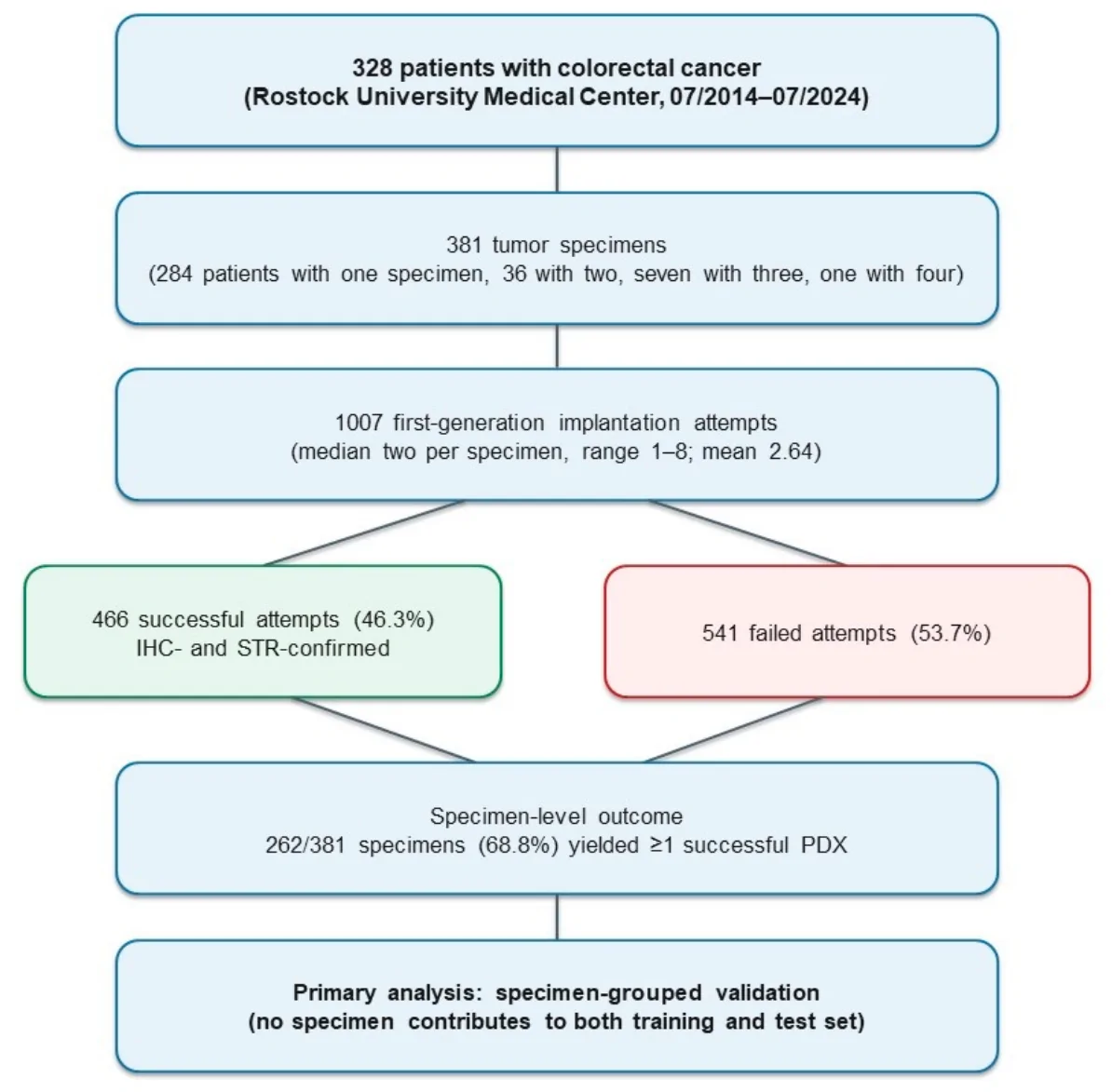
Patient, specimen and attempt flow. Derivation of the analysis set from 328 patients through 381 tumor specimens to 1007 implantation attempts, showing attempt-level and specimen-level outcomes and the unit of the primary grouped analysis.

A total of 328 patients contributed 381 tumor specimens, from which 1007 first-generation PDX establishment attempts were performed (Figure 2). Of the 328 patients, 284 contributed one specimen, 36 contributed two, seven contributed three, and one contributed four. The number of attempts per specimen ranged from one to eight (mean 2.64, median 2); 254 specimens underwent exactly two attempts and 106 specimens underwent four or more. Repeat attempts were performed when a specimen was considered of particular value and a model was specifically desired (for example, Lynch syndrome or young-onset cases) or when an initial attempt was lost to a technical failure. Attempts on a given specimen were performed sequentially rather than concurrently, as viably cryopreserved fragments allowed for later re-implantation; attempts from the same specimen are therefore not statistically independent and are treated as clustered throughout. Clinical and pathological characteristics of the 1007 attempts are summarized in Table 1 and the experimental characteristics are summarized in Table 2. The complete dataset used for model development is provided in Supplementary Table S2.

### 3.2. Engraftment Outcomes and the Clustered Structure of the Data

Of 1007 attempts, 466 were successful, corresponding to an attempt-level engraftment rate of 46.3%. At the specimen level, 262 of 381 specimens (68.8%) yielded at least one successful first-generation model, whereas 119 (31.2%) failed across all attempts.

The outcomes were clearly correlated within specimens: the exchangeable within-specimen correlation estimated by generalized estimating equations was 0.31. This correlation is substantial but far from complete. Among the 254 specimens implanted exactly twice, both attempts succeeded in 87, both failed in 87, and the two attempts yielded discordant results in 80 (31.5%). Nearly a third of specimens therefore produced both a success and a failure under nominally identical conditions, which places an empirical ceiling on the accuracy attainable by any model that uses only specimen-level information.

### 3.3. Primary Analysis: Specimen-Grouped Validation

**Figure 3:**
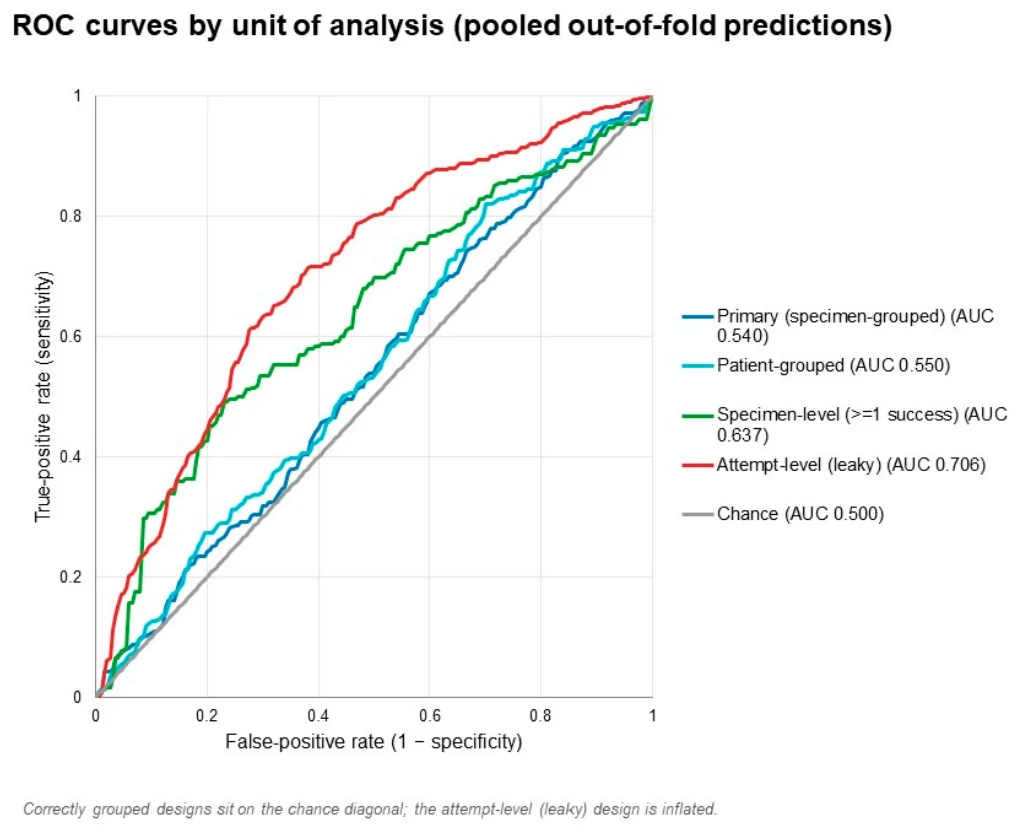
Receiver operating characteristic curves under three validation designs. Curves are computed from pooled out-of-fold predictions; shaded bands are 95% cluster (specimen) bootstrap intervals. The near-coincidence of the two attempt-level curves shows that preprocessing leakage contributes negligibly to the inflation relative to specimen-grouped validation.

Under specimen-grouped partitioning with a fully leakage-free, nested pipeline, the mean test AUC across resampling iterations was 0.538 (2.5th–97.5th percentile 0.437–0.619). Pooling out-of-fold predictions gave an AUC of 0.550 with a cluster-bootstrap 95% confidence interval of 0.508–0.591 (Figure 3). The interval excludes 0.50, so discrimination is above chance, but only marginally so; the lower bound corresponds to performance that would be indistinguishable from random ranking in routine use.

**Figure 4:**
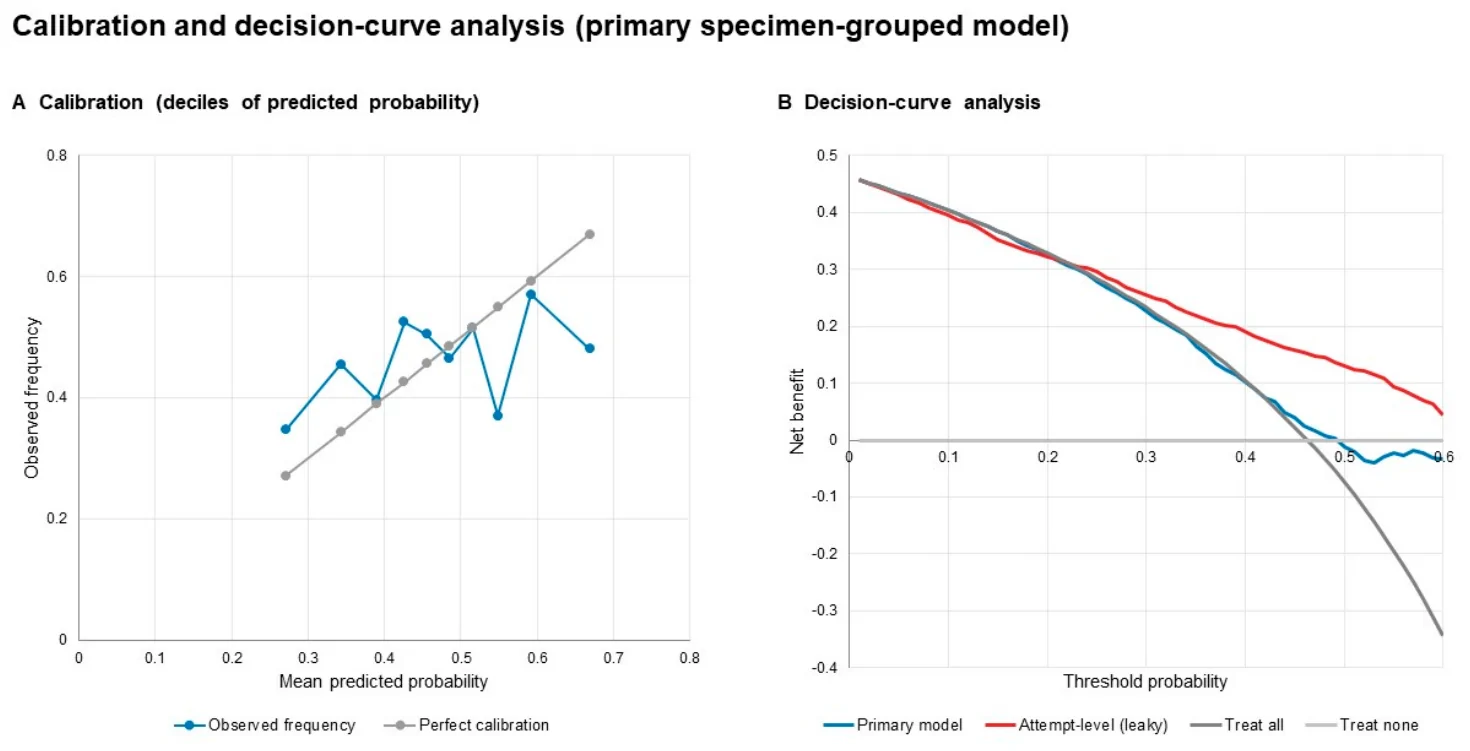
Calibration and decision curve analysis under specimen-grouped validation. (**A**) Observed proportion of successful attempts against mean predicted probability by decile of predicted risk for pooled out-of-fold predictions. (**B**) Net benefit of the specimen-grouped model compared with implanting all specimens and implanting none across threshold probabilities.

Precision–recall performance was 0.500 against an outcome prevalence of 0.463, an improvement of under four percentage points over the no-information baseline. The Brier score was 0.253, essentially identical to the 0.249 obtained by predicting the overall engraftment rate for every attempt. At a probability threshold of 0.5, the sensitivity was 0.419, specificity was 0.641, positive predictive value was 0.503, negative predictive value was 0.561 and accuracy was 0.538. No alternative operating point yielded a clinically useful balance, the Youden-optimal threshold giving sensitivity and specificity that still traded off around a near-chance ranking. Calibration was acceptable in the middle of the predicted-risk range, but calibration was weakest in the extreme deciles of predicted risk, consistent with a model whose predictions are compressed toward the base rate (Figure 4).

The result was not attributable to the choice of learner. Under identical specimen-grouped validation, penalized logistic regression and a random forest using all predictors without feature selection both achieved comparable near-chance discrimination (mean AUC within 0.02 of the primary estimate). Three learners of very different flexibility therefore converge on the same conclusion.

### 3.4. Patient-Grouped Validation

Because 44 patients contributed more than one specimen, a patient-grouped analysis was performed so that all specimens and attempts from a given patient remained together. The mean test AUC was 0.549 (0.473–0.610), with a pooled out-of-fold AUC of 0.548 (95% CI 0.502–0.592). Patient-level dependence beyond specimen-level dependence therefore has little additional influence, as expected given that most patients contributed a single specimen.

### 3.5. Effect of the Unit of Analysis and of Preprocessing Leakage

**Figure 5:**
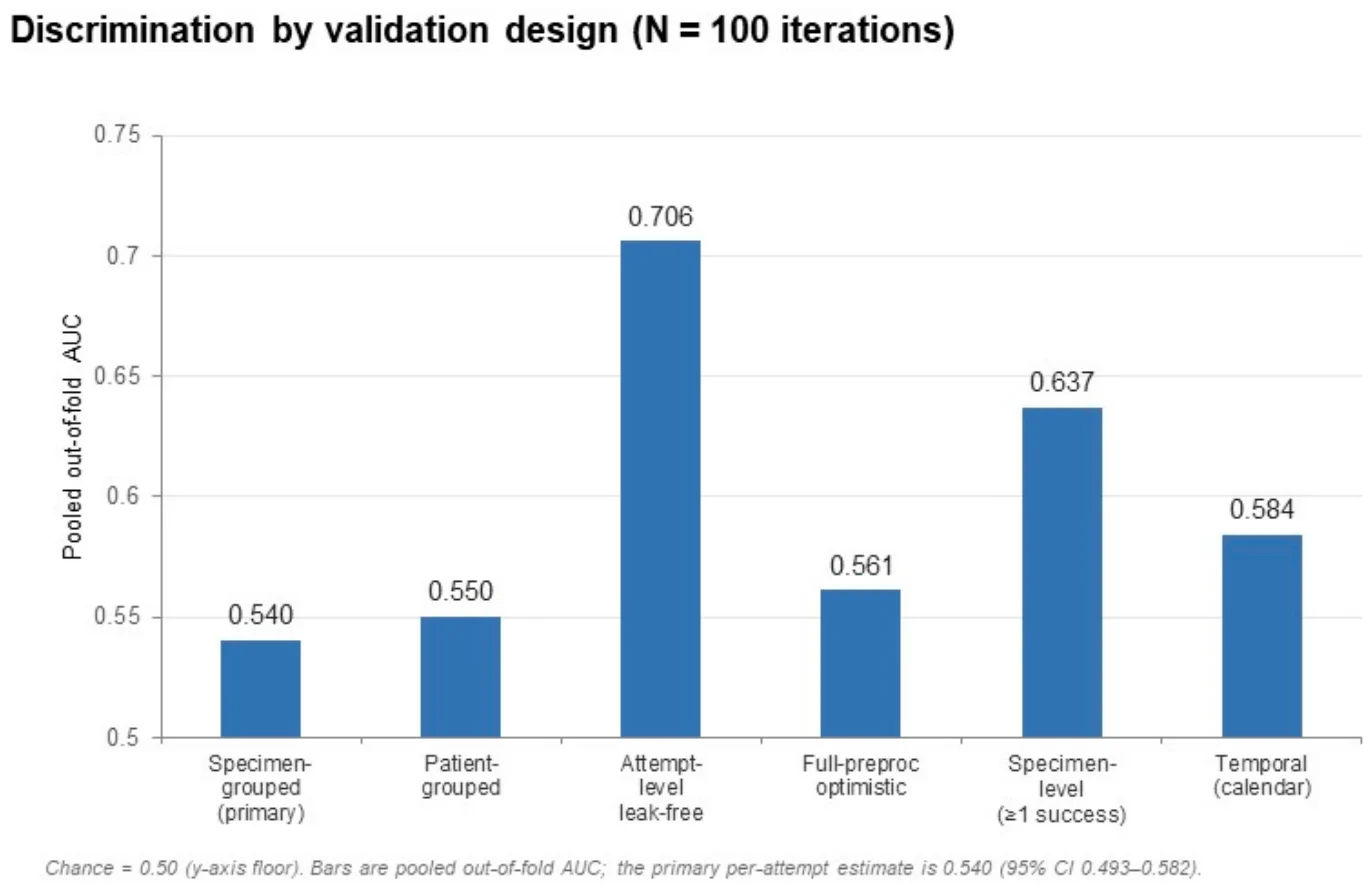
Discrimination under different validation designs. Mean test AUC with 2.5th–97.5th percentile intervals across resampling iterations for five validation designs applied to the identical dataset and modeling pipeline. The dotted line marks chance performance.

Holding the pipeline constant and varying only the partitioning unit produced a large and systematic difference in apparent performance (Figure 5). Random attempt-level splitting with the same leakage-free nested pipeline yielded a mean test AUC of 0.684 (0.631–0.728), and attempt-level splitting with imputation, standardization and feature selection performed once on the full dataset yielded 0.691 (0.632–0.741).

The near identity of these two estimates is itself informative. Preprocessing leakage, the mechanism most often invoked in methodological critiques of clinical machine learning, accounted for essentially none of the inflation in this dataset—under 0.01 AUC. The entire gap between roughly 0.69 and 0.55 is attributable to the presence of attempts from the same tumor on both sides of the split. When a specimen contributes several attempts and those attempts share every patient- and tumor-level covariate, a flexible model can identify the specimen from its covariate signature and reproduce the outcomes it has already seen for that specimen.

### 3.6. Specimen-Level Prediction

When the analysis was moved to the specimen level, with the 381 specimens as units, the outcome defined as whether a specimen produced at least one first-generation model, the number of attempts included as a predictor, and partitioning grouped by patient, the mean test AUC was 0.628 (0.532–0.718) and the pooled out-of-fold AUC was 0.636. Precision–recall performance was 0.777 against a prevalence of 0.688.

This estimate exceeds the attempt-level grouped estimate, but the comparison is not a like-for-like improvement in predictive signal. The specimen-level outcome is markedly less balanced, and a substantial part of the discrimination is carried by the number of attempts performed: specimens implanted more often are more likely to yield at least one model. At a 0.5 threshold, the specimen-level model classified 96.6% of specimens as likely to succeed, achieving a specificity of 0.034. Only at the Youden-optimal threshold of 0.72 did it become discriminating (sensitivity 0.466, specificity 0.807, positive predictive value 0.841), and at that operating point, it would decline nearly half of the specimens that in fact went on to produce a usable model.

### 3.7. Temporal Validation

To assess whether a model transfers across calendar time, each specimen was assigned as a whole unit to an earlier or a later period according to its median surgery date (cut-off October 2016), so that no specimen contributed attempts to both periods. A model trained on the earlier period (500 attempts from 192 specimens, engraftment rate 45.4%) and tested on the later period (507 attempts from 189 specimens, engraftment rate 47.1%) achieved a test AUC of 0.584 (95% CI 0.520–0.650), a PR-AUC of 0.528 and a Brier score of 0.262. The confidence interval excludes 0.50, and the estimate coincides with the specimen-grouped primary analysis (0.550). A model fitted on earlier surgeries therefore transferred to later ones with the same marginal discrimination observed throughout, indicating that calendar-time evolution of the program does not conceal a stronger underlying signal. Procedural practice did evolve over the series but not to a designed schedule: Matrigel^®^, adopted after we observed improved take rates and retained thereafter, and host strain, which shifted with animal availability, both varied in frequency across the recorded early- and late-experience strata (χ^2^ *p* < 0.001 for each; these reflect changes in default practice rather than controlled comparisons). Fresh tissue was used rarely and to a similar extent throughout (6.2% of attempts), consistent with our previous finding of no significant loss of engraftment with cryopreservation [20]; logistical constraints, not outcome, governed that choice.

### 3.8. Sensitivity Analyses

Restricting the analysis to the 995 attempts involving adenocarcinoma, thereby excluding the 12 neuroendocrine tumors, left the specimen-grouped estimate essentially unchanged (within 0.02 of the primary). Alternative handling of missing values in grade, lymphatic invasion, vascular invasion and MSI status—treating missingness as an explicit category, and a complete-case analysis restricted to attempts with no missing values in these four variables—likewise altered the pooled AUC by no more than approximately 0.02. No strategy materially changed the conclusion, and the direction of the small differences argues against missingness masking a real signal. Early deaths were infrequent and did not drive the result: 24 attempts (2.4%) ended within 30 days of implantation, of which 23 were failures and one a success. Recoding all 24 as failures, or excluding them entirely, left the specimen-grouped AUC essentially unchanged (change < 0.01 in both cases).

### 3.9. Stability of Feature Selection and Cluster-Robust Associations

**Figure 6:**
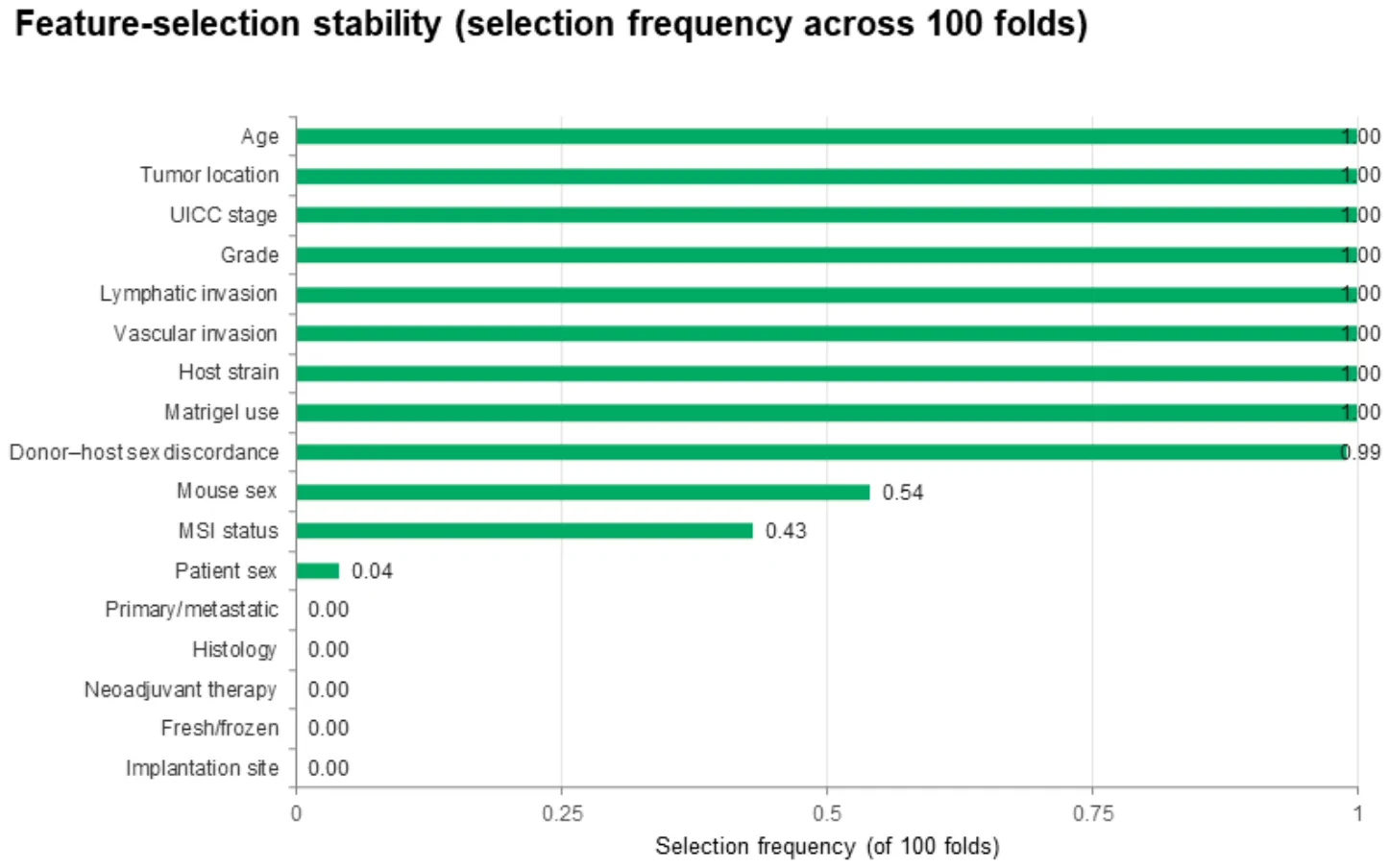
Stability of feature selection under specimen-grouped validation. Proportion of the 100 specimen-grouped resampling iterations in which each variable was retained by random-forest importance filtering.

Across the 100 specimen-grouped resampling iterations, eight variables were retained in essentially every iteration—patient age, anatomical site, UICC stage, grade, host strain, Matrigel use, lymphatic invasion, and vascular invasion (selection frequency ≈ 1.00), together with donor–host sex discordance (0.97)—while MSI status was retained in 67% of iterations (Figure 6). High selection stability in the presence of near-chance discrimination indicates that the filter reproducibly ranks the same correlated variables, not that those variables carry decisive information.

Cluster-robust univariable associations at the attempt level identified MSI-H status (OR 1.99, 95% CI 1.33–2.99, *p* = 0.0009), higher UICC stage (OR 1.26 per stage, 1.09–1.46, *p* = 0.002), metastatic or recurrent rather than primary origin (OR 1.62, 1.09–2.43, *p* = 0.018) and adenocarcinoma rather than neuroendocrine histology (OR 11.8 for adenocarcinoma, equivalently OR 0.085 for neuroendocrine tumors, 0.015–0.486, *p* = 0.006) as being associated with engraftment. In a multivariable model restricted to attempts with complete pathology, only MSI-H status retained an independent association (OR 1.90, 1.15–3.14, *p* = 0.012); the estimate for peritoneal and abdominal-organ specimens was unstable owing to near-separation (10 of 14 attempts successful) and should not be interpreted. Notably, none of the experimental variables—host strain, Matrigel use, fresh versus cryopreserved tissue, host sex, donor–host sex discordance or experimental period—showed a significant association after accounting for clustering.

These associations are reported as descriptive and predictive associations rather than biological or causal determinants. Several of the variables are strongly intercorrelated—anatomical site with primary versus metastatic origin, UICC stage with both—so the attribution of a shared association to any one of them is arbitrary. The apparent conflict between statistically detectable associations and near-chance discrimination is not a contradiction: an odds ratio of 2 for a single binary variable, in a setting where the outcome is close to a coin flip, shifts predicted probabilities too little to separate individual attempts.

### 3.10. Subgroup Analyses

Engraftment differed across clinically defined subgroups. MSI-H tumors were engrafted in 59.2% of attempts versus 44.2% for MSS/MSI-L tumors; UICC stage I and stage II specimens were engrafted in 40.0% and 40.1% of attempts versus 49.8% and 50.8% for stages III and IV; metastatic or recurrent specimens were engrafted in 54.3% versus 44.2% for primary tumors. Differences across experimental variables were smaller: NMRI nude 47.1%, NSG 45.1%, NOD SCID 43.5%; Matrigel 47.8% versus 44.2% without; fresh tissue 50.0% versus cryopreserved 46.0%; early-experience stratum 47.9% versus late-experience stratum 44.6%.

Discrimination within subgroups, computed from the primary grouped out-of-fold predictions, was uniformly poor and inconsistent: MSS/MSI-L 0.517 versus MSI-H 0.455, UICC I 0.501, II 0.522, III 0.623, and IV 0.486, and primary 0.571 versus metastatic 0.373. Several subgroups fell at or below 0.50. The limited overall performance is therefore not explained by a well-predicted subgroup being diluted by a poorly predicted one; no subgroup is predicted well.

## 4. Discussion

The central finding of this study is negative and, we would argue, useful: for a previously unseen colorectal tumor, routinely available clinicopathological and experimental variables do not meaningfully predict whether an implantation attempt will produce a PDX model. Under specimen-grouped validation, the pooled AUC was 0.550 (95% CI 0.508–0.591), sensitivity and specificity at a conventional threshold were 0.42 and 0.64, and precision–recall performance barely exceeded the no-information baseline. These numbers do not support the triage of specimens, selection of host strain, or allocation of the number of implantation attempts on the basis of routine data.

The methodological observation underlying this conclusion is that the unit of analysis, not preprocessing hygiene, governs the result. In this dataset, eliminating preprocessing leakage changed the attempt-level estimate by less than 0.01 AUC, whereas holding whole specimens out reduced it from roughly 0.69 to 0.55. This ordering matters for how the literature on PDX engraftment prediction should be read. Reports of AUCs in the 0.7–0.8 range derived from datasets in which one tumor contributes several implantations, split at random, are not necessarily reporting a predictive signal; they may be reporting the model’s ability to recognize tumors it has already seen. The remedy is not more careful scaling or imputation but a change in the partitioning unit, and we would encourage grouped validation to be treated as the default rather than as a sensitivity analysis in this field.

Three independent lines of evidence within this study converge on the same limit. Calendar-time temporal validation, holding whole specimens to one side of the split, gave 0.584 (95% CI 0.520–0.650), coinciding with the specimen-grouped primary estimate. Subgroup discrimination was poor in every subgroup examined, with several subgroups at or below 0.50. And the discordance of bilateral implantations provides a direct empirical bound: among specimens implanted exactly twice, 31.5% produced one success and one failure.

Why the routine variables fail is biologically unsurprising. Engraftment depends on the viability of the implanted fragment, on ischemia time between resection and implantation, on the proportion of tumor cells relative to stroma and necrosis within the specific 3 mm fragment used, on stromal and immune composition, on extracellular-matrix support, and on the stochastic survival of a small number of tumor-initiating cells [22,23,24,25]. None of these factors are captured by UICC stage or tumor grade, and several vary between fragments taken from the same tumor, which is precisely the source of within-specimen discordance observed here. The associations that did survive cluster-robust modeling—MSI-H status, advanced stage, and metastatic origin—are consistent with more aggressive tumor biology conferring a modest advantage, and MSI-H engraftment advantage has been described previously; however, an odds ratio near 2 for a single variable is far from sufficient for individual-level prediction when the base rate is close to 50%.

Several limitations should be considered. First, this is a retrospective single-center study; institutional protocols, tissue handling and host strains may limit generalizability, and no external validation cohort was available. It is worth noting, however, that the direction of the principal finding is unlikely to be reversed by external data: a negative result obtained under conservative validation is more robust to institutional idiosyncrasy than a positive one. Second, key technical covariates known to affect engraftment—cold-ischemia time, tumor-cell viability, and content in the implanted fragment, as well as operator-level detail—were not recorded and could not be evaluated. The ceiling we describe is therefore a ceiling on prediction from routinely collected variables and should not be read as a claim that engraftment is biologically unpredictable. Third, the classifier and feature-selection strategy carried into the grouped analyses were chosen in an attempt-level screen; we mitigated this by confirming the result with penalized logistic regression and with an unfiltered random forest, both of which gave comparable estimates, but a fully grouped model-selection procedure over all 90 combinations was not computationally undertaken. Fourth, only binary engraftment was modeled; latency to palpable tumor and subsequent growth rate are informative outcomes that were not systematically recorded across the full ten-year period and represent a natural extension of this work. Fifth, MSI status was missing in 20.3% of attempts, and although four imputation strategies yielded concordant results, informative missingness cannot be excluded.

The practical implication for PDX programs is to stop looking for a triage rule in routine data and to record, prospectively, the variables that plausibly carry the signal: ischemia time, fragment-level tumor cellularity and necrosis, quantitative viability of the implanted tissue, and molecular characterization of the donor tumor. A prospective study collecting these alongside engraftment outcome, analyzed with grouped validation from the outset, would be able to establish whether the ceiling described here is a property of the data currently collected or of the biology itself.

## 5. Conclusions

In 1007 first-generation CRC-PDX establishment attempts from 381 specimens and 328 patients, routinely available clinicopathological and experimental variables provided only marginal discrimination of engraftment when whole tumors were held out of model training (pooled AUC 0.550, 95% CI 0.508–0.591). The substantially higher performance obtained under attempt-level random splitting reflected the repeated sampling of individual tumors rather than a generalizable predictive signal and was almost entirely unaffected by preprocessing leakage. Nearly a third of the twice-implanted specimens yielded discordant outcomes, placing an empirical bound on specimen-level prediction. The prediction of CRC-PDX engraftment that is sufficient to guide practice will require the prospective collection of tissue-quality and molecular variables that are not part of routine pathology, evaluated from the outset under grouped validation.

## Figures and Tables

**Table 1:** Clinical and pathological characteristics of tumor samples in PDX model establishment.

Variables	
Total number of experiments (*n*)	1007
Age of patients at tumor donation (years, mean ± SD)	69.4 ± 12.7
Patient Gender (*n*, %)	
Male	619 (61.5)
Female	388 (38.5)
Tumor Type (*n*, %)	
Adenocarcinoma	995 (98.8)
Neuroendocrine tumor	12 (1.2)
Tumor Location (*n*, %)	
Right colon	383 (38.0)
Transverse	65 (6.5)
Left colon	92 (9.1)
Sigmoid	171 (17.0)
Rectum	98 (9.7)
Brain (confirmed CRC brain metastasis)	4 (0.4)
Lung	27 (2.7)
Liver	153 (15.2)
Peritoneum and Abdominal Organs	14 (1.4)
Pathological Tumor Stage (*n*, %)	
I	110 (10.9)
II	292 (29.0)
III	207 (20.6)
IV	398 (39.5)
Tumor Grade (*n*, %)	
G1	37 (3.7)
G2	581 (57.7)
G3/4	264 (26.2)
Unknown	125 (12.4)
Lymphatic Invasion (Yes/No/Unknown)	238/637/132
Vascular Invasion (Yes/No/Unknown)	383/488/136
MSI Status (*n*, %)	
MSS/MSI-L	629 (62.5)
MSI-H	174 (17.3)
unknown	204 (20.3)
Received Neoadjuvant Therapy before Specimen Collection (Yes/No)	75/932
Sample origin	
Primary	799(79.3)
Metastatic/recurrent tumors	208(20.7)

**Table 2:** Information about the mice and experimental procedures in PDX model establishment.

Variables	
Total number of experiments (*n*)	1007
Mouse Strain (*n*, %)	
NOD SCID	23 (2.28)
NSG	379 (37.64)
NMRI nu/nu	605 (60.08)
Mouse Sex (*n*, %)	
Female	684 (67.92)
Male	323 (32.08)
Tumor Implantation Site (*n*, %)	
Left Sides	27 (2.68)
Both Sides	980 (97.32)
Sex-discordant host (Yes/No) *	542/465
Sample type (*n*, %)	
Fresh	62 (6.16)
Frozen	945 (93.84)
Experience stratum (*n*, %)	
Late experience	504 (50.05)
Early experience	503 (49.95)
Matrigel (Yes/No)	582/425

* Sex-discordant host: implantation of tumor tissue from a donor of one sex into a mouse of the opposite sex.

## Data Availability

The complete attempt-level dataset underlying all analyses is provided as Supplementary Table S2, including specimen and patient identifiers. Analysis code, package versions, model hyperparameters and random seeds are available as supplementary data.

## Supplementary Materials

The following supporting information can be downloaded at: https://www.mdpi.com/article/10.3390/cancers18182996/s1, Supplementary Table S1. Summary of missing data percentages. Supplementary Table S2. Complete attempt-level dataset of 1007 CRC-PDX establishment attempts, including specimen and patient identifiers. File S1. python script.

## Abbreviations

AUC, area under the receiver operating characteristic curve; CRC, colorectal cancer; GEE, generalized estimating equations; MSI, microsatellite instability; PDX, patient-derived xenograft; PR-AUC, area under the precision–recall curve; STR, short tandem repeat; UICC, Union for International Cancer Control.
